# Asylum-seekers in Germany differ from regularly insured in their morbidity, utilizations and costs of care

**DOI:** 10.1371/journal.pone.0197881

**Published:** 2018-05-24

**Authors:** Sebastian Bauhoff, Dirk Göpffarth

**Affiliations:** 1 Center for Global Development, Washington, D.C., United States of America; 2 State Chancellery of North Rhine-Westphalia, Düsseldorf, Germany; Medizinische Universitat Wien, AUSTRIA

## Abstract

In the wake of the European refugee crisis, Germany has received over a million new applications for asylum in the last two years. The health care system is struggling to provide asylum-seekers with access to essential medical services and facilitate their longer-term integration. In this article, we report on the morbidity, utilization and costs of care for a sample of asylum-seekers as compared to a matched group of regularly insured. Using administrative data, we found that asylum-seekers had more hospital and emergency department admissions, including more admissions that could be avoided through good outpatient care or prevention. Their average expenditures were 10 percent higher than for the regularly insured, mostly because of higher hospital expenditures, although there was substantial variation in expenditures by country of origin. Facilitating access to the health care system, especially outpatient and mental health care, could improve asylum-seekers health status and integration, possibly at lower costs.

## Introduction

Germany is struggling to integrate the recent influx of migrants, many of whom apply for asylum. This is also a challenge to the health care system. The wave of arrivals is unprecedented: since early 2015, Germany has received 1.3 million first-time applications for asylum, about 1.6 percent of the country’s population and more than twice as many as in the preceding decade [1].

Refugees and asylum-seekers are granted access to medical care services by international law and, in the case of Germany, also by European Union directives and the German Constitution. Beyond these legal requirements, providing good access to the regular health care system can help ameliorate acute, chronic and preventable conditions, and thereby improve individuals’ health status, lower medium-term health care spending, and facilitate overall integration [2,3].

There is limited evidence on the needs, utilization and costs of providing care for the most recent group of arrivals. The majority of recent arrivals comes from Syria, Afghanistan or Iraq and are relatively young and predominantly male [1]. Their health issues vary and diagnoses are often not specific, although pain and respiratory infections are common diagnoses [4]. The prevalence of communicable diseases is relatively low and may be associated with post-migratory conditions in Germany [4,5]. Estimates of mental health issues, including post-traumatic stress disorders, vary and likely under-estimate true prevalence [4,6,7]. Language barriers, bureaucracy and administrative restrictions are thought to hinder access and–ultimately–lead to unnecessary costs as asylum-seekers receive avoidable emergency and hospital care [7,8].

Although informative, these patterns are difficult to interpret without data on a group of regular users of the health care system with comparable demographic profiles. A benchmarked and more nuanced comparison could more effectively inform policy action, as well as the public discourse regarding the costs of accepting refugees in Germany and elsewhere, including the United States.

We examined the morbidity, utilization and costs of care for a sample of asylum-seekers in Germany in 2016. We drew on routine data from a large health plan tasked with administering claims and reimbursements for several municipalities hosting asylum-seekers. We compared the findings for our sample with a group of regularly insured with similar age, gender and place of residence. We also conducted subgroup analyses by the asylum-seekers country of origin.

### Policy context

In Germany, there are three stages of access to health care services for asylum-seekers [9]. Initially, centralized reception centers in the German states provide shelter, health assessments and basic health services; they also process the application for asylum. After 6–12 weeks, most asylum-seekers are relocated to municipalities that are responsible for providing basic benefits, including access to a limited set of health care services. Finally, individuals whose application has been accepted, who are still awaiting a decision after 15 months, or are awaiting expulsion have access to the same health care benefits as German citizens.

The limited benefits available to asylum-seekers during the waiting period cover treatments for pain and acute conditions (or chronic conditions that could become acute if not treated), and emergency and maternity care. Public health services, such as immunizations, and translation services are also available. Entitlement to services outside the limited benefits is authorized on a case-by-case basis by the municipalities [10]. Access is also restricted and asylum-seekers are generally required to obtain prior agreement for non-emergency care from the respective municipality. However, municipalities can contract with health plans that, in turn, issue a standard electronic health card that grants direct access to primary and specialist outpatient care, as well as inpatient services. Other services, such as mental health care, dental prostheses and rehabilitations still require explicit approval. So far, only a few municipalities have chosen to make use of the electronic health card [11]. Whereas German health plans have some (limited) opportunities to manage the care of the regularly insured, they are mostly processing payments for the asylum-seekers on behalf of the municipality.

## Study data and methods

### Data sources

We obtained data on asylum-seekers and regularly insured individuals from the BARMER health plan, which has been contracted by several municipalities to administer payments for asylum-seekers awaiting a decision on their application. Both groups use electronic health cards that employ the same routine processes to generate data on diagnoses, utilization, prescriptions and expenditures.

The data originating from outpatient care and dentists span the first through third quarter of 2016; all other data span the full year. We also observe individuals’ place of residence, as well as their gender and age, as categorized by the 20 gender-age groups that are used in the German health plan payment system. The BARMER health plan provided permissions to use these data.

## Methods

Our analytical sample covers all 3,639 asylum-seekers who have received an electronic health card and were assigned to BARMER for at least one day in 2016. About two-thirds (62 percent) of the sample is male, about one-third (34 percent) is younger than 18, and 70 percent are below the age of 30 (Table 1). Although there are 45 origin countries in our sample, more than half of individuals come from just three countries, Syria (23 percent), Afghanistan (18 percent) and Iraq (14 percent). National level data on first-time applicants for asylum in 2016 show comparable gender and age distributions, and the top-3 origin countries are the same as in our sample (S1 Appendix). However, due to the contracting process, our sample is geographically concentrated: 97% live in three towns and one district in two states, Schleswig-Holstein and North Rhine Westphalia.

**Table 1:** Age-gender distribution and top origin countries for the sample of asylum-seekers.

**Age distribution**	**All**	**Female**		**Male**	
	**N**	**N**		**N**	
Younger than 18	1,232	546	44%	686	56%
18–29	1,302	379	29%	923	71%
30–39	666	256	38%	410	62%
Older than 39	439	187	43%	252	57%
All	3,639	1,368	38%	2,271	62%
**Origin countries**	**Individuals**	**Insured-days**		**Share in sample**	
Syria	838	129,060		23%	
Afghanistan	669	137,202		18%	
Iraq	527	91,992		14%	
Albania	198	31,620		5%	
Armenia	194	34,877		5%	
Iran	159	30,391		4%	
Eritrea	135	22,697		4%	
Macedonia	125	21,109		3%	
Serbia	121	18,707		3%	
Kosovo	94	12,845		3%	
Russian Federation	89	12,161		2%	
Others	579	83,708		16%	
All	3,639	614,208			

Source: Author calculations.

We constructed a matched comparison group of 18,191 individuals who were regularly insured through the BARMER health plan in 2016. For each asylum-seeker, we identified the set of regularly insured individuals who live in the same area and fall into the same gender-age group. We randomly selected five matches for each asylum seeker out of an average of 100 possible matches (S2 Appendix); in one case we could only find a single match. We only use data from the matched comparisons that were generated in the same period as that for the asylum-seeker; the average coverage period is 169 days.

We investigated patterns in diagnoses, utilization and expenditures. We calculated annual expenditures for inpatient, outpatient and dental care, as well as drugs and other items, such as durable medical equipment. We extrapolated the three quarters of expenditure data for outpatient and dental care to annual figures. We constructed prevalence rates for the diagnoses for each chapter of the International Classification of Diseases (ICD) and health care utilization. For these measures, we report annual prevalence rates except for outpatient and dental care, where we report on the three quarters of available data. Finally, we calculated the prevalence of hospital admissions for Ambulatory Care Sensitive Conditions (ACSC). ACSC are conditions for which the risk of hospitalization can be considerably reduced by effective treatment, good management or immunization. In Germany, a list of relevant ACSC has been identified by group consensus methods among a panel of 40 physicians from all medical disciplines [12].

In supplemental analyses, we disaggregated the data using three groupings of country of origin. The first group consists of 2,328 asylum-seekers from Syria, Afghanistan, Iraq, Iran and Eritrea who are afforded a high rate of protection of 50 percent or more (98 percent for Syrians in 2016). These individuals face a relatively high likelihood that their application will be accepted and otherwise a low risk of deportation. The second group covers 565 individuals from the Western Balkan states, such as Albania, Macedonia and Serbia. Asylum-seekers from these countries generally have a low rate of protection. The third group includes 746 individuals from a diverse set of other countries, predominantly asylum-seekers from Armenia and the Russian Federation. We directly standardized expenditures of each group to the age-gender distribution of the overall sample of asylum-seekers; we indirectly standardized the morbidity and utilization figures because of the small sample sizes. We also conducted more detailed case studies of three conditions, acute inflammation of the mucous membranes, hypertension and psychological disorders. Standardization is a common statistical method to mitigate the effects of differences in age or other confounding variables when comparing two or more variables. This can be effected by either referring to a standard population (direct method) or a set of (age-)specific rates (indirect method) [13].

Our findings are subject to several limitations. First, our sample is small, and it is limited to several municipalities that are working with the BARMER plan and are using the electronic health card. Other municipalities may work with other insurers, such as regional health plans, or administer the benefits themselves. Second, we use administrative data generated when individuals interact with the health care system; we do not observe true morbidity. Third, although our research design compares individuals with similar age-gender profiles, we lack objective benchmarks, e.g., to assess whether there is true under or over-use of certain services. However, the prevalence of ambulatory care sensitive conditions and the findings from the three case studies provide some indications in this regard. Fourth, the time periods of our outpatient and inpatient data do not quite align, with the former covering only the first three quarters of 2016. Finally, the sample is too small to conduct a full heterogeneity analysis across the heterogeneous set of origin countries. Our grouping by the rate of protection is coarse but policy-relevant as it is correlated with the likelihood of staying in Germany and thus remaining in the health care system.

## Study results

### Morbidity

Based on the combined outpatient and inpatient diagnoses, codes that represent unclassified factors influencing health status and contact with health services providers (ICD chapter 21) were the most prevalent among asylum-seekers and the matched comparison group (Table 2, left column). The relatively higher prevalence of these codes for unspecific symptoms and factors among asylum-seekers (671 compared to 566 per 1,000 for the regularly insured) may indicate particular sociodemographic needs of this group. Within this ICD chapter, codes for health services related to reproduction were common in both groups, but asylum-seeker had almost twice as many encounters for health hazards relating to communicable disease, while the regularly insured had a higher rate of preventive examinations.

**Table 2:** Diagnoses (per 1,000 insured).

ICD chapter		Combined outpatient and hospital diagnoses*		Principal Hospital diagnosis*	
#	Title	Asylum-seekers	Matched Comparison	Asylum-seekers	Matched Comparison
1	Certain infectious and parasitic diseases	137	134	5.8	0.8
2	Neoplasms	60	65	4.7	1.9
3	Diseases of the blood and blood-forming organs and certain disorders involving the immune mechanism	46	36	2.5	0.1
4	Endocrine, nutritional and metabolic diseases	185	263	1.4	1.9
5	Mental and behavioral disorders	271	395	13.2	5.1
6	Diseases of the nervous system	85	108	5.0	4.1
7	Diseases of the eye and adnexa	165	217	3.3	3.0
8	Diseases of the ear and mastoid process	76	85	3.6	0.9
9	Diseases of the circulatory system	143	153	11.3	2.9
10	Diseases of the respiratory system	279	361	9.9	6.8
11	Diseases of the digestive system	190	148	13.2	5.9
12	Diseases of the skin and subcutaneous tissue	150	160	5.0	2.6
13	Diseases of the musculoskeletal system and connective tissue	322	379	12.4	2.6
14	Diseases of the genitourinary system	209	202	6.6	3.0
15	Pregnancy, childbirth and the puerperium	318	119	34.9	14.6
16	Certain conditions originating in the perinatal period	31	15	5.8	1.3
17	Congenital malformations, deformations and chromosomal abnormalities	35	86	2.8	3.1
18	Symptoms, signs and abnormal clinical and laboratory findings, not elsewhere classified	323	240	15.7	4.0
19	Injury, poisoning and certain other consequences of external causes	154	165	8.2	4.9
21	Factors influencing health status and contact with health services	671	566	14.0	7.8

Source: Author calculations.

Note

* Outpatient diagnoses for quarters 1–3 of 2016; hospital diagnoses for all quarters of 2016.

Codes for pregnancy and perinatal problems were more than twice as common among asylum-seekers, with a prevalence of 318 per 1,000 insured compared to 119 among the regularly insured. This reflects a higher birth rate among asylum-seekers as the rate of deliveries in this group was 3.5 times higher but also a rate of abortive outcomes that was 6.8 times higher than for the matched comparison group.

In line with prior research [4], infectious and parasitic diseases were not more prevalent among asylum-seekers than in the comparison group. However, tuberculosis is far more common with a prevalence of 3.6 per 1,000 compared to 0.1 among the regularly insured. These figures are not directly comparable as asylum-seekers are subject to compulsory screening. Furthermore, hepatitis was far more widespread among asylum-seekers with a prevalence of 11.5 per 1,000, compared to 2.0.

Mental health issues are coded more often among regular insured in the comparison group than among asylum-seekers, possibly because of massive underreporting of mental health problems of asylum-seekers [14]. The ICD-group level analysis shows that schizophrenic, schizotypal, delusional and affective disorders are relatively more commonly diagnosed among asylum-seekers (S3 Appendix).

Some but not all chronic diseases were strikingly widespread among asylum-seekers. This was the case with nutritional anemia (which could have been acquired during flight) with a prevalence of 19.5 per 1,000 (compared to 8.9), a common disease among refugees [15]. Other chronic diseases were as prevalent among asylum-seekers as in the comparison group, such as hypertension (prevalence of 61.6 and 61.7, respectively) or diabetes (48.1 and 50.0). Obesity, on the other hand was far less frequent (28.6 and 46.0).

### Utilization

As a group, asylum-seekers had about twice as many inpatient encounters, emergency department admissions and admissions with mental health diagnoses in the observation period when compared to the matched comparison (Table 3). One in eight asylum-seekers had a hospital admission compared to one in twenty in the comparison group. The shorter average length of stay could indicate that asylum-seekers seek hospital care for lower-severity issues.

**Table 3:** Utilization.

Indicator	Asylum-seekers	Matched Comparison
*Inpatient care*		
All hospitalizations (per 1,000 insured)	179	77
Admissions for Ambulatory Care Sensitive Conditions (per 1,000)	38	13
Emergency department (per 1,000 insured)	88	31
Admissions for Ambulatory Care Sensitive Conditions (per 1,000)	21	8
Individuals with 1 or more hospitalizations (per 1,000 insured)	121	48
Hospital days (per 1,000 insured)	860	468
Average length of stay	4.8	6.1
Individuals with 1 or more hospitalizations related to mental health (per 1,000 insured)	8	4
Outpatient care*		
Outpatient encounters (per 1,000 insured)	848	889
Individuals with 1 or more outpatient encounters (per 1,000 insured)	402	429
Average outpatient encounters, for those who had 1 or more encounters	2.1	2.1
Primary care physician encounters (per 1,000 insured)	315	300
Specialist encounters (per 1,000 insured)	374	410
Psychotherapy encounters (per 1,000 insured)	6	20
*Drugs and medical equipment*		
Prescriptions (per 1,000 insured)	1,551	2,729
Insured with 1 or more prescriptions (per 1,000 insured)	482	543
Average prescriptions per insured for those with 1 or more prescriptions	3.2	5
Prescribed medical equipment (per 1,000 insured)	1	411
Dental care*		
Dental encounters (per 1,000 insured)	296	870
Dental encounters for prostheses (per 1,000 insured)	8	47
Individuals with 1 or more dental encounters (per 1,000 insured)	215	424
Average dental encounters for those with 1 or more dental encounters	1.4	2.1

Source: Author calculations.Note: The above figures are not annual because the length of the underlying insurance spells may be shorter. However, the columns are comparable because the spells for each match are identical.

* Data for outpatient and dental care are for quarters 1–3 of 2016 only.

The causes of hospitalizations are reflected in the reported principal hospital diagnoses (Table 2, right column). About 20% of the higher utilization can be explained by pregnancies, deliveries and the puerperium. A high portion of the remaining cases can be ascribed to unspecific symptoms and factors such as abdominal pain, headache or back pain.

Asylum-seekers were also more likely to be admitted for conditions that could have been avoided through good outpatient care or prevention. About 21 percent of hospital admissions were for ambulatory care sensitive conditions, compared to about 18 percent for the matched comparison. The share of these conditions in emergency department admissions is comparable for the two groups.

Utilization of primary care physicians was comparable for both groups but asylum-seekers had fewer specialist and substantially fewer outpatient mental health encounters. This suggests substantial under-provision of these services in the outpatient setting, also in light of the documented psychological stresses for this group and the comparatively higher inpatient admissions for mental health. The mandatory prior authorization for outpatient mental health visits may represent an important barrier to access, alongside with language and cultural barriers. This requirement applies to all insured but may be more difficult to navigate for asylum-seekers.

Although asylum-seekers were only slightly less likely to have at least one prescription, the average number of prescriptions was about one-third lower than for the comparison group of regularly insured (3.2 compared to 5.0). Medical equipment was rarely prescribed for asylum-seekers and they were half as likely to have had any dental encounter, despite evidence of relatively worse dental health. Policy-induced barriers may contribute also to these patterns as medical equipment and prosthesis require prior authorization.

### Expenditures

**Fig 1:**
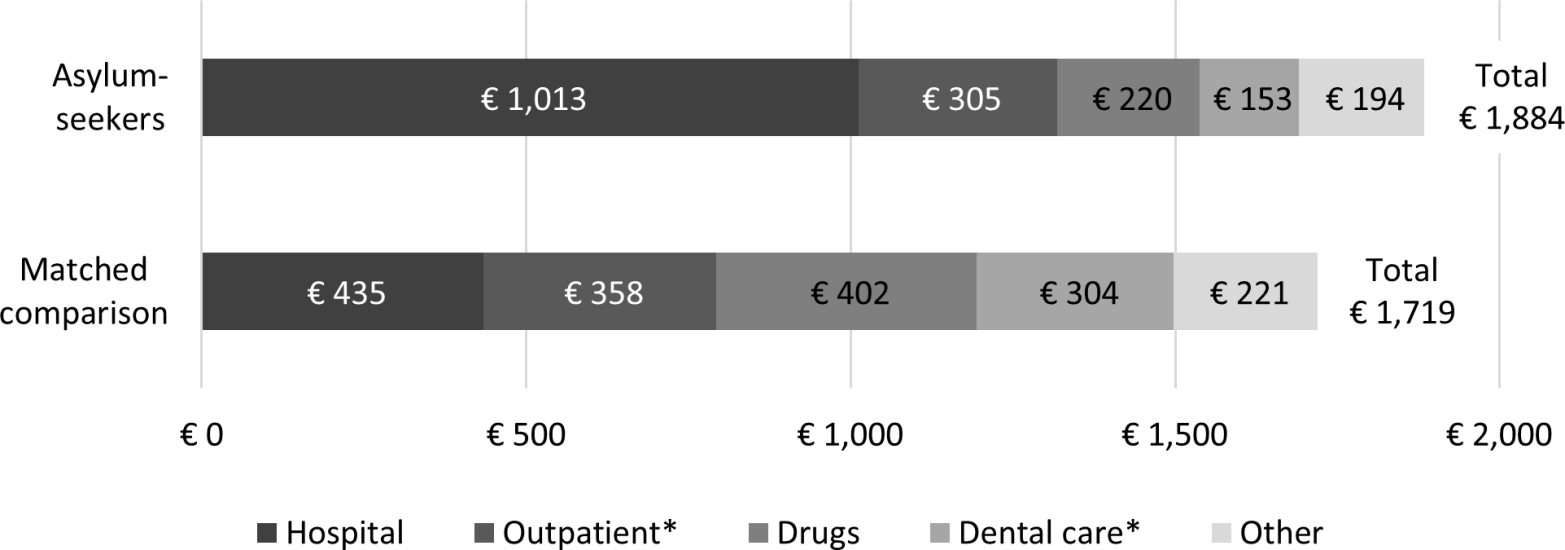
Average expenditures. Source: Author calculations. Note: * Outpatient and dental care expenditures extrapolated from Q1-Q3 of 2016 to full year.

The average total expenditures were 10 percent higher for asylum-seekers than for the regularly insured, about €1,884 compared to €1,719 (USD2,005 and USD1,829 at 2016 exchange rates; Fig 1). The diverging patterns of morbidity and utilization were reflected in the composition of expenditures. In particular, hospital expenditures were twice as high for asylum-seekers, whereas expenditures for drugs and dental care were about half those of the comparison group. Expenditures for outpatient care were about 15 percent lower for asylum-seekers.

### Heterogeneity among the asylum-seekers

While our overall sample of asylum-seekers differs from the matched comparison of regularly insured, there were also differences within the former (S4 Appendix).

In terms of morbidity and utilization, asylum-seekers from Syria, Afghanistan, Iraq, Iran and Eritrea (country group A) have higher birth rates, but otherwise generally a lower morbidity. They had lower hospital utilization and fewer prescriptions than applicants from other countries. Those from the Western Balkan states (country group B) have a higher morbidity, even after controlling for age and sex. Compared to the other groups, asylum-seekers from these countries are comparatively often coded with mental health problems and diseases of the circulatory and nervous system. Individuals in this group also had comparatively higher inpatient and outpatient encounters for mental health.

The overall composition of expenditures also differed across the country groupings. Individuals from country group A had lower average expenditures (€1,596) than individuals in the other groups and the regularly insured. Their hospital expenditures were also lower relative to the other groups of asylum-seekers, in particular those from the heterogeneous group of “other” countries whose average hospital costs were more than three times higher than those for the overall matched comparison. We cannot further disaggregate this group because of limited sample sizes.

### Case studies of three conditions

The broad pattern of relatively higher hospital expenditures, fewer prescriptions and lower drug expenditures for asylum-seekers also holds for three specific conditions we investigated (S5 Appendix): acute inflammation of the mucous membranes, hypertension and psychological disorders. Asylum-seekers had a higher prevalence of these conditions and, for each diagnosed case, their average hospital expenditures were substantially higher (between 1.3 times for psychological disorders and 3 times for hypertension) compared to the matched group of regularly insured, while drug expenditures were between 21 (hypertension) and 80 (acute infections) percent lower.

## Discussion

Ensuring that asylum-seekers have adequate access to medical care remains a priority and challenge for Germany’s health care system. We found that the recent wave of asylum-seekers has different morbidity, utilization and cost profiles than a matched comparison group of regularly insured. In particular, asylum-seekers and the comparison group shared the most important (by prevalence) disease categories but the prevalences were quite different. Diagnoses for pregnancy and childbirth were relatively more prevalent in this group, both overall and for hospital admissions. Asylum-seekers had more than twice as many inpatient and emergency department encounters, but fewer prescriptions and dental visits. These differences in utilization translated into similar differences in expenditure patterns. Although the average total expenditures for asylum-seekers were 10 percent higher than for the comparison group, the hospital expenditures were twice as high while expenditures for other categories were lower.

Several of our findings suggest that better access to primary care could help reduce the relatively higher use of hospital and emergency care, and the higher rate of ambulatory care sensitive conditions. It could also improve the early management of chronic conditions, as our three case studies suggest. Similarly, improving access to outpatient mental health care services could improve health outcomes and–by reducing mental health related hospitalizations–may reduce expenditures. One concrete way of achieving these improvements could be early screening for mental health issues in this high-risk population, outreach through community health workers, as well as assistance in overcoming language barriers and navigating the health care system. Experiences from the city-state of Bremen suggest that such efforts could be effective, although this approach may be constrained by factor within and outside the health care system, e.g., limited availability of psychiatric services and adequate living quarters, and barriers to broader integration [4]. General outreach and assistance could also help increase familiarity with and use of primary care options.

There is also scope to improve the design, administration and management of health care services for asylum-seekers. Restricting access to otherwise available services may increase rather than decrease costs, as utilization shifts to inpatient care and preventable conditions are not treated or managed [7]. Many municipalities still do not use the standard electronic health card, so that patients need prior authorization from a government agency rather than physicians. Early experiences in some German states suggest that the electronic health card reduces access barriers and administrative costs [16,17], although the limited benefits would remain a constraint, e.g., for mental health services. Similarly, the contracted health plans could be allowed and incentivized to improve the management of patient care rather than merely administering payments for the municipalities. This could also facilitate the transition to the regular membership in the Social Health Insurance.

More generally, encouraging local experimentation and rigorous evaluation of how to provide cost-effective access to care during the waiting period could yield substantial value. Further research is also warranted with regards to the heterogeneity among the asylum-seekers and how best to work with individuals with different demographic profiles (including age and gender), from very diverse backgrounds and with diverse health issues. Similarly, future studies could examine in greater detail detailed diagnoses, e.g., for mental health. In addition, it is valuable to track the asylum-seekers’ transition to full membership in the Social Health Insurance and longer-term experiences with the health care system. Individuals in our study sample had filed for asylum at most 15 months previously, as they would otherwise would have graduated to be fully insured. Studies of longer-term migrants in Germany indicate a continued over-reliance on emergency care and under-use of preventive care, frequent switching of primary care providers, low health literacy, and different conceptions of illness and the health care system [18,19].

Our analysis also has bearing on the debate in the United States regarding the costs and benefits of accepting refugees–including those from Syria, Afghanistan and Iraq, who are disproportionally represented in our study. Unlike immigrants, refugees arriving in the United States are granted access to medical care services, e.g., through temporary membership in state Medicaid or the Refugee Medical Assistance programs. Several current estimates of the costs of accepting refugees into the U.S. assume that their health care spending is equivalent the average costs of programs such as Medicare and Medicaid [20,21]; our analysis suggests that there may be substantial heterogeneity within this group. As in Germany, there is evidence of barriers to access in the U.S. [22] and promise in learning from local initiatives that work with providers and refugees to improve coordination and facilitate access [23].

## Conclusion

Managing the large influx of refugees and asylum-seekers is a major challenge for Germany in all aspects, including providing access to social services, housing and the labor market. Providing effective access to the health care system is critical to successful integration, as well as a moral and legal obligation. Our study points to several ways to improve this process to the benefit of individuals–and possibly at lower cost. To achieve these improvements, Germany–and other receiving countries–should enable and encourage municipalities to adapt promising approaches, as well as study innovative local pilots. Given that the recent large flows of refugees may recur in the future, there is an urgent need for better health policy toward these vulnerable populations.

## Supporting information

S1 Appendix Characteristics of first time asylum-seekers at the national level. (DOCX)

S2 Appendix Matching approach. (DOCX)

S3 Appendix Morbidity by ICD group. (DOCX)

S4 Appendix Morbidity, utilization and expenditures by asylum-seekers’ country-group. (DOCX)

S5 Appendix Selected case studies. (DOCX)
