# Supplementary material for: Asylum-seekers in Germany differ from regularly insured in their morbidity, utilizations and costs of care
Source: PLoS One. 2018 May 24;13(5):e0197881. doi: 10.1371/journal.pone.0197881 (PMC5967831; doi:10.1371/journal.pone.0197881)
Supplement: S1 Appendix — (DOCX) [file pone.0197881.s001.docx]

**S1 Appendix: Characteristics of first-time asylum-seekers at the national level**

**Table S1.1. Age-gender distribution and top-10 origin countries of first-time applicants for asylum at the national level (2016)**

| **Age distribution** | **All** | **Female** | | **Male** | |  |
| --- | --- | --- | --- | --- | --- | --- |
|  | **N** | **N** |  | **N** |  |  |
| Younger than 18 | 261,386 | 106,853 | 41% | 154,533 | 59% |  |
| 18-29 | 271,413 | 70,402 | 26% | 201,011 | 74% |  |
| 30-39 | 114,952 | 40,120 | 35% | 74,832 | 65% |  |
| Older than 39 | 74,619 | 30,429 | 41% | 44,190 | 59% |  |
| All | 722,370 | 247,804 | 34% | 474,566 | 66% |  |
|  |  |  | |  | | |
| **Origin countries** | **Individuals** |  | | **Share** | | |
| Syria | 266,250 |  | | 37% | | |
| Afghanistan | 127,012 |  | | 18% | | |
| Iraq | 96,116 |  | | 13% | | |
| Iran | 26,426 |  | | 4% | | |
| Eritrea | 18,854 |  | | 3% | | |
| Albania | 14,853 |  | | 2% | | |
| Unknown | 14,659 |  | | 2% | | |
| Pakistan | 14,484 |  | | 2% | | |
| Nigeria | 12,709 |  | | 2% | | |
| Russian Federation | 10,985 |  | | 2% | | |
| Other | 120,022 |  | | 17% | | |
| **All** | **722,370** |  | |  | | |

*Source:* (1)
