# Supplementary material for: Asylum-seekers in Germany differ from regularly insured in their morbidity, utilizations and costs of care
Source: PLoS One. 2018 May 24;13(5):e0197881. doi: 10.1371/journal.pone.0197881 (PMC5967831; doi:10.1371/journal.pone.0197881)
Supplement: S2 Appendix — (DOCX) [file pone.0197881.s002.docx]

# S2 Appendix: Matching approach

Each asylum-seeker is matched to five insured (1:5 match). The matching criteria are:

- Gender
- Age group
- Geographic location (state-district)

The potential matches must be in the same cells and be continuously insured over the same period as the asylum-seeker.

We used gender-age groups used by the German risk adjustment system. In our data, there are a total of 159 gender-age-locality cells. In most cases there is a large number of potential matches and the actual matches are chosen at random.

Figure B.1 shows the distribution of the share of actual matches in the potential matches and Table B.1 shows the average share across all localities, by gender-age group. The median share across the age-gender-location cells is 0.04.

For 150 of the cells (94%) there are 10 or more potential matches for each asylum-seeker. For one cell, the number of actual matches equals the number of potential matches. For one other cell, there was only one potential match. Four cases could not be matched and were excluded from the analysis.

We only use those diagnoses, utilization and expenditure data from the matched observations that are collected in the exact same time period as the data for the asylum-seeker.

**Figure S2.1: Distribution of the share of actual matches in potential matches**
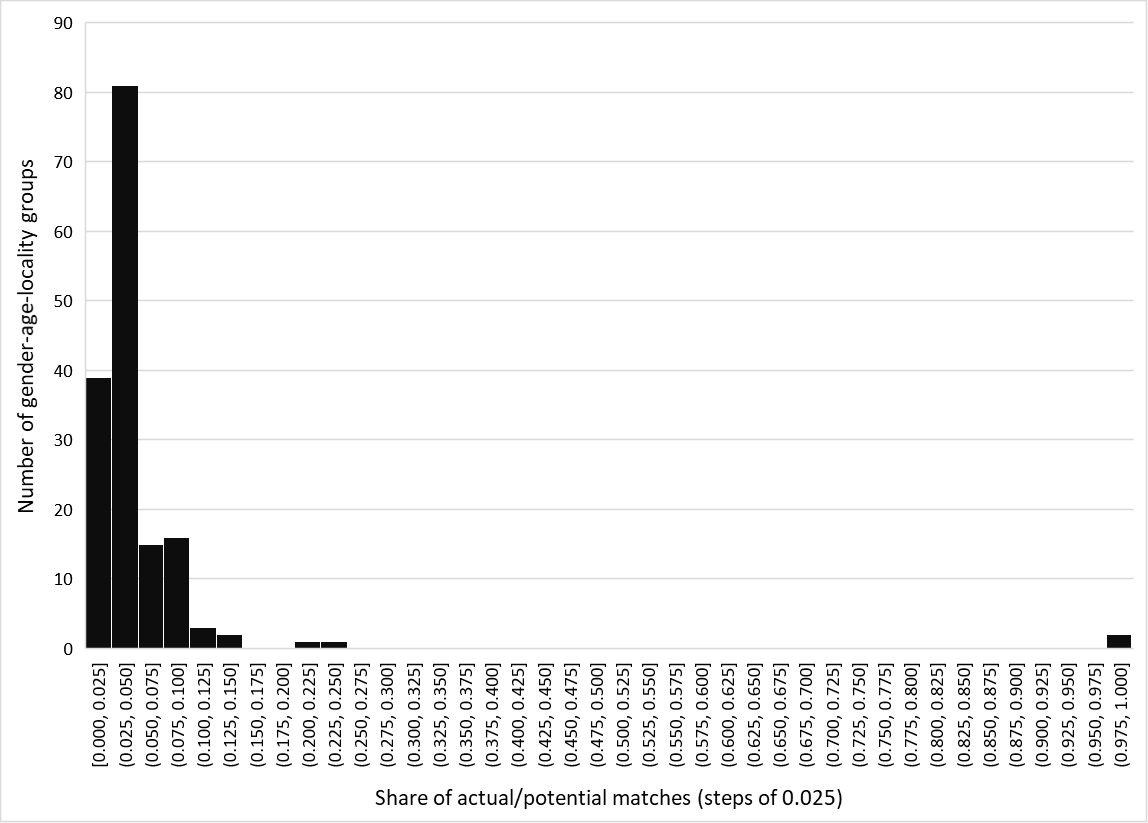


**Table S2.1: Average share of actual matches in potential matches (across all localities)**

| **Age group** | **All** | **Female** | **Male** |
| --- | --- | --- | --- |
| 0 | 0.34 | 0.14 | 0.53 |
| 1-5 | 0.06 | 0.06 | 0.06 |
| 6-12 | 0.05 | 0.05 | 0.05 |
| 13-17 | 0.05 | 0.04 | 0.05 |
| 18-24 | 0.05 | 0.05 | 0.04 |
| 25-29 | 0.04 | 0.04 | 0.04 |
| 30-34 | 0.04 | 0.04 | 0.04 |
| 35-39 | 0.04 | 0.04 | 0.04 |
| 40-44 | 0.03 | 0.04 | 0.03 |
| 45-49 | 0.02 | 0.02 | 0.03 |
| 50-54 | 0.02 | 0.02 | 0.02 |
| 55-59 | 0.02 | 0.02 | 0.02 |
| 60-64 | 0.03 | 0.02 | 0.04 |
| 65-69 | 0.04 | 0.03 | 0.04 |
| 70-74 | 0.04 | 0.03 | 0.04 |
| 75-79 | 0.02 | 0.02 | n/a |
| 80-84 | 0.03 | 0.03 | 0.03 |
| **All ages** | **0.05** | **0.04** | **0.06** |
