# Supplementary material for: Asylum-seekers in Germany differ from regularly insured in their morbidity, utilizations and costs of care
Source: PLoS One. 2018 May 24;13(5):e0197881. doi: 10.1371/journal.pone.0197881 (PMC5967831; doi:10.1371/journal.pone.0197881)
Supplement: S3 Appendix — (DOCX) [file pone.0197881.s003.docx]

# S3 Appendix: Morbidity by ICD Group

# Table S3.1: Morbidity by ICD Group (prevalence per 1,000 insured)

| **From** | **To** | **ICD Group** | **Asylum-Seekers** | **Matched Comparison** |
| --- | --- | --- | --- | --- |
| A00 | A09 | Intestinal infectious diseases | 18.1 | 34.4 |
| A15 | A19 | Tuberculosis | 3.6 | 0.1 |
| A20 | A28 | Certain zoonotic bacterial diseases | 0.5 | 0.4 |
| A30 | A49 | Other bacterial diseases | 4.9 | 6.1 |
| A50 | A64 | Infections with a predominantly sexual mode of transmission | 1.4 | 0.4 |
| A65 | A69 | Other spirochaetal diseases | 0.3 | 0.6 |
| A70 | A74 | Other diseases caused by chlamydiae | 0.3 | 0.0 |
| A92 | A99 | Arthropod-borne viral fevers and viral haemorrhagic fevers | 0.3 | 0.6 |
| B00 | B09 | Viral infections characterized by skin and mucous membrane lesions | 12.4 | 19.4 |
| B15 | B19 | Viral hepatitis | 11.5 | 2.0 |
| B20 | B24 | Human immunodeficiency virus [HIV] disease | 0.0 | 1.4 |
| B25 | B34 | Other viral diseases | 16.5 | 30.9 |
| B35 | B49 | Mycoses | 30.2 | 17.8 |
| B65 | B83 | Helminthiases | 4.7 | 1.2 |
| B85 | B89 | Pediculosis, acariasis and other infestations | 6.0 | 2.4 |
| B90 | B94 | Sequelae of infectious and parasitic diseases | 0.3 | 0.0 |
| B95 | B98 | Bacterial, viral and other infectious agents | 16.2 | 4.3 |
| B99 | B99 | Other infectious diseases | 9.9 | 11.7 |
| C00 | C14 | Malignant neoplasms of lip, oral cavity and pharynx | 0.0 | 0.7 |
| C15 | C26 | Malignant neoplasms of digestive organs | 4.4 | 2.0 |
| C30 | C39 | Malignant neoplasms of respiratory and intrathoracic organs | 0.0 | 0.4 |
| C40 | C41 | Malignant neoplasms of bone and articular cartilage | 0.0 | 0.1 |
| C43 | C44 | Melanoma and other malignant neoplasms of skin | 0.0 | 1.3 |
| C50 | C50 | Malignant neoplasm of breast | 5.2 | 3.4 |
| C51 | C58 | Malignant neoplasms of female genital organs | 0.0 | 0.5 |
| C60 | C63 | Malignant neoplasms of male genital organs | 0.5 | 1.9 |
| C64 | C68 | Malignant neoplasms of urinary tract | 2.2 | 0.2 |
| C69 | C72 | Malignant neoplasms of eye, brain and other parts of central nervous system | 0.5 | 0.0 |
| C73 | C75 | Malignant neoplasms of thyroid and other endocrine glands | 0.0 | 0.3 |
| C76 | C80 | Malignant neoplasms of ill-defined, secondary and unspecified sites | 11.0 | 3.0 |
| C81 | C96 | Malignant neoplasms, stated or presumed to be primary, of lymphoid, haematopoietic and related tissue | 2.7 | 1.8 |
| C97 | C97 | Malignant neoplasms of independent (primary) multiple sites | 0.0 | 0.2 |
| D00 | D09 | In situ neoplasms | 0.0 | 0.9 |
| D10 | D36 | Benign neoplasms | 29.7 | 43.2 |
| D37 | D48 | Neoplasms of uncertain or unknown behaviour | 4.1 | 5.2 |
| D50 | D53 | Nutritional anaemias | 19.5 | 8.9 |
| D55 | D59 | Haemolytic anaemias | 5.8 | 1.9 |
| D60 | D64 | Aplastic and other anaemias | 12.9 | 6.4 |
| D65 | D69 | Coagulation defects, purpura and other haemorrhagic conditions | 3.8 | 6.9 |
| D70 | D77 | Other diseases of blood and blood-forming organs | 2.5 | 1.5 |
| D80 | D90 | Certain disorders involving the immune mechanism | 1.1 | 10.1 |
| E00 | E07 | Disorders of thyroid gland | 34.1 | 77.3 |
| E10 | E14 | Diabetes mellitus | 48.1 | 51.0 |
| E15 | E16 | Other disorders of glucose regulation and pancreatic internal secretion | 0.3 | 0.1 |
| E20 | E35 | Disorders of other endocrine glands | 6.0 | 7.2 |
| E40 | E46 | Malnutrition | 1.6 | 0.7 |
| E50 | E64 | Other nutritional deficiencies | 10.2 | 9.2 |
| E65 | E68 | Obesity and other hyperalimentation | 28.6 | 46.0 |
| E70 | E90 | Metabolic disorders | 55.8 | 71.5 |
| F00 | F09 | Organic, including symptomatic, mental disorders | 2.2 | 3.2 |
| F10 | F19 | Mental and behavioural disorders due to psychoactive substance use | 17.0 | 28.5 |
| F20 | F29 | Schizophrenia, schizotypal and delusional disorders | 12.9 | 10.9 |
| F30 | F39 | Mood [affective] disorders | 83.8 | 69.5 |
| F40 | F48 | Neurotic, stress-related and somatoform disorders | 104.1 | 125.1 |
| F50 | F59 | Behavioural syndromes associated with physiological disturbances and physical factors | 13.7 | 21.1 |
| F60 | F69 | Disorders of adult personality and behaviour | 2.7 | 22.8 |
| F70 | F79 | Mental retardation | 2.5 | 8.2 |
| F80 | F89 | Disorders of psychological development | 20.9 | 62.0 |
| F90 | F98 | Behavioural and emotional disorders with onset usually occurring in childhood and adolescence | 9.3 | 40.6 |
| F99 | F99 | Unspecified mental disorder | 1.4 | 3.0 |
| G00 | G09 | Inflammatory diseases of the central nervous system | 0.0 | 2.4 |
| G20 | G26 | Extrapyramidal and movement disorders | 1.9 | 4.4 |
| G30 | G32 | Other degenerative diseases of the nervous system | 0.0 | 0.1 |
| G35 | G37 | Demyelinating diseases of the central nervous system | 1.1 | 9.7 |
| G40 | G47 | Episodic and paroxysmal disorders | 58.3 | 56.1 |
| G50 | G59 | Nerve, nerve root and plexus disorders | 11.8 | 15.6 |
| G60 | G64 | Polyneuropathies and other disorders of the peripheral nervous system | 1.1 | 7.8 |
| G70 | G73 | Diseases of myoneural junction and muscle | 0.8 | 0.2 |
| G80 | G83 | Cerebral palsy and other paralytic syndromes | 5.8 | 8.5 |
| G90 | G99 | Other disorders of the nervous system | 4.4 | 3.2 |
| H00 | H06 | Disorders of eyelid, lacrimal system and orbit | 8.0 | 11.3 |
| H10 | H13 | Disorders of conjunctiva | 18.7 | 21.4 |
| H15 | H22 | Disorders of sclera, cornea, iris and ciliary body | 1.6 | 3.4 |
| H25 | H28 | Disorders of lens | 9.6 | 2.4 |
| H30 | H36 | Disorders of choroid and retina | 6.0 | 9.3 |
| H40 | H42 | Glaucoma | 4.1 | 4.6 |
| H43 | H45 | Disorders of vitreous body and globe | 0.8 | 1.4 |
| H46 | H48 | Disorders of optic nerve and visual pathways | 0.5 | 3.1 |
| H49 | H52 | Disorders of ocular muscles, binocular movement, accommodation and refraction | 95.1 | 139.2 |
| H53 | H54 | Visual disturbances and blindness | 17.3 | 19.5 |
| H55 | H59 | Other disorders of eye and adnexa | 2.7 | 1.2 |
| H60 | H62 | Diseases of external ear | 22.5 | 28.9 |
| H65 | H75 | Diseases of middle ear and mastoid | 23.1 | 25.4 |
| H80 | H83 | Diseases of inner ear | 2.5 | 1.5 |
| H90 | H95 | Other disorders of ear | 27.8 | 29.4 |
| I00 | I02 | Acute rheumatic fever | 0.3 | 0.0 |
| I05 | I09 | Chronic rheumatic heart diseases | 1.4 | 0.5 |
| I10 | I15 | Hypertensive diseases | 61.6 | 61.7 |
| I20 | I25 | Ischaemic heart diseases | 25.6 | 9.6 |
| I26 | I28 | Pulmonary heart disease and diseases of pulmonary circulation | 6.6 | 1.6 |
| I30 | I52 | Other forms of heart disease | 22.8 | 33.4 |
| I60 | I69 | Cerebrovascular diseases | 3.6 | 4.8 |
| I70 | I79 | Diseases of arteries, arterioles and capillaries | 3.3 | 5.4 |
| I80 | I89 | Diseases of veins, lymphatic vessels and lymph nodes, not elsewhere classified | 9.1 | 27.8 |
| I95 | I99 | Other and unspecified disorders of the circulatory system | 8.5 | 8.0 |
| J00 | J06 | Acute upper respiratory infections | 133.0 | 133.4 |
| J09 | J18 | Influenza and pneumonia | 6.3 | 6.0 |
| J20 | J22 | Other acute lower respiratory infections | 17.9 | 26.1 |
| J30 | J39 | Other diseases of upper respiratory tract | 55.5 | 115.2 |
| J40 | J47 | Chronic lower respiratory diseases | 44.8 | 65.5 |
| J60 | J70 | Lung diseases due to external agents | 0.3 | 0.1 |
| J80 | J84 | Other respiratory diseases principally affecting the interstitium | 0.5 | 0.9 |
| J90 | J94 | Other diseases of pleura | 1.4 | 0.2 |
| J95 | J99 | Other diseases of the respiratory system | 19.0 | 13.1 |
| K00 | K14 | Diseases of oral cavity, salivary glands and jaws | 37.9 | 14.0 |
| K20 | K31 | Diseases of oesophagus, stomach and duodenum | 73.9 | 34.2 |
| K35 | K38 | Diseases of appendix | 5.2 | 4.9 |
| K40 | K46 | Hernia | 8.0 | 9.5 |
| K50 | K52 | Noninfective enteritis and colitis | 8.0 | 34.0 |
| K55 | K64 | Other diseases of intestines | 35.7 | 28.6 |
| K65 | K67 | Diseases of peritoneum | 3.8 | 1.4 |
| K70 | K77 | Diseases of liver | 8.0 | 8.1 |
| K80 | K87 | Disorders of gallbladder, biliary tract and pancreas | 4.9 | 9.5 |
| K90 | K93 | Other diseases of the digestive system | 4.4 | 3.7 |
| L00 | L08 | Infections of the skin and subcutaneous tissue | 16.2 | 12.3 |
| L10 | L14 | Bullous disorders | 0.8 | 0.1 |
| L20 | L30 | Dermatitis and eczema | 57.7 | 65.2 |
| L40 | L45 | Papulosquamous disorders | 8.2 | 23.0 |
| L50 | L54 | Urticaria and erythema | 4.1 | 5.1 |
| L55 | L59 | Radiation-related disorders of the skin and subcutaneous tissue | 0.8 | 1.7 |
| L60 | L75 | Disorders of skin appendages | 40.7 | 41.8 |
| L80 | L99 | Other disorders of the skin and subcutaneous tissue | 21.4 | 10.5 |
| M05 | M14 | Inflammatory polyarthropathies | 8.0 | 7.3 |
| M15 | M19 | Arthrosis | 15.1 | 19.4 |
| M20 | M25 | Other joint disorders | 59.1 | 52.2 |
| M30 | M36 | Systemic connective tissue disorders | 2.5 | 3.2 |
| M40 | M43 | Deforming dorsopathies | 13.5 | 27.7 |
| M45 | M49 | Spondylopathies | 16.2 | 26.1 |
| M50 | M54 | Other dorsopathies | 132.5 | 123.1 |
| M60 | M63 | Disorders of muscles | 8.2 | 23.6 |
| M65 | M68 | Disorders of synovium and tendon | 4.7 | 7.4 |
| M70 | M79 | Other soft tissue disorders | 37.4 | 48.8 |
| M80 | M85 | Disorders of bone density and structure | 3.3 | 1.9 |
| M86 | M90 | Other osteopathies | 4.1 | 1.5 |
| M91 | M94 | Chondropathies | 2.7 | 3.4 |
| M95 | M99 | Other disorders of the musculoskeletal system and connective tissue | 14.8 | 33.9 |
| N00 | N08 | Glomerular diseases | 3.3 | 1.6 |
| N10 | N16 | Renal tubulo-interstitial diseases | 2.7 | 4.9 |
| N17 | N19 | Renal failure | 12.9 | 8.0 |
| N20 | N23 | Urolithiasis | 11.8 | 3.1 |
| N25 | N29 | Other disorders of kidney and ureter | 1.6 | 2.5 |
| N30 | N39 | Other diseases of urinary system | 30.2 | 31.4 |
| N40 | N51 | Diseases of male genital organs | 24.7 | 12.3 |
| N60 | N64 | Disorders of breast | 6.3 | 4.2 |
| N70 | N77 | Inflammatory diseases of female pelvic organs | 13.5 | 6.7 |
| N80 | N98 | Noninflammatory disorders of female genital tract | 101.1 | 127.2 |
| N99 | N99 | Other disorders of the genitourinary system | 0.3 | 0.1 |
| O00 | O08 | Pregnancy with abortive outcome | 6.3 | 0.9 |
| O09 | O09 | Length of pregnancy | 113.8 | 40.8 |
| O10 | O16 | Oedema, proteinuria and hypertensive disorders in pregnancy, childbirth and the puerperium | 1.9 | 1.8 |
| O20 | O29 | Other maternal disorders predominantly related to pregnancy | 44.8 | 20.0 |
| O30 | O48 | Maternal care related to the fetus and amniotic cavity and possible delivery problems | 61.6 | 21.3 |
| O60 | O75 | Complications of labour and delivery | 33.5 | 13.9 |
| O80 | O82 | Delivery | 18.1 | 5.1 |
| O85 | O92 | Complications predominantly related to the puerperium | 5.8 | 3.2 |
| O94 | O99 | Other obstetric conditions, not elsewhere classified | 32.4 | 11.8 |
| P00 | P04 | Fetus and newborn affected by maternal factors and by complications of pregnancy, labour and delivery | 0.0 | 0.7 |
| P05 | P08 | Disorders related to length of gestation and fetal growth | 9.6 | 5.8 |
| P10 | P15 | Birth trauma | 0.3 | 0.1 |
| P20 | P29 | Respiratory and cardiovascular disorders specific to the perinatal period | 6.6 | 3.1 |
| P35 | P39 | Infections specific to the perinatal period | 1.1 | 0.3 |
| P50 | P61 | Haemorrhagic and haematological disorders of fetus and newborn | 5.5 | 0.1 |
| P70 | P74 | Transitory endocrine and metabolic disorders specific to fetus and newborn | 0.5 | 2.1 |
| P75 | P78 | Digestive system disorders of fetus and newborn | 0.0 | 0.1 |
| P80 | P83 | Conditions involving the integument and temperature regulation of fetus and newborn | 1.9 | 0.7 |
| P90 | P96 | Other disorders originating in the perinatal period | 4.9 | 2.4 |
| Q00 | Q07 | Congenital malformations of the nervous system | 1.4 | 1.3 |
| Q10 | Q18 | Congenital malformations of eye, ear, face and neck | 0.3 | 4.9 |
| Q20 | Q28 | Congenital malformations of the circulatory system | 4.1 | 16.7 |
| Q30 | Q34 | Congenital malformations of the respiratory system | 0.0 | 1.1 |
| Q35 | Q37 | Cleft lip and cleft palate | 1.6 | 1.9 |
| Q38 | Q45 | Other congenital malformations of the digestive system | 0.8 | 0.9 |
| Q50 | Q56 | Congenital malformations of genital organs | 3.8 | 3.8 |
| Q60 | Q64 | Congenital malformations of the urinary system | 3.0 | 4.8 |
| Q65 | Q79 | Congenital malformations and deformations of the musculoskeletal system | 14.6 | 39.9 |
| Q80 | Q89 | Other congenital malformations | 5.5 | 9.2 |
| Q90 | Q99 | Chromosomal abnormalities, not elsewhere classified | 0.0 | 1.8 |
| R00 | R09 | Symptoms and signs involving the circulatory and respiratory systems | 51.7 | 41.6 |
| R10 | R19 | Symptoms and signs involving the digestive system and abdomen | 102.2 | 47.6 |
| R20 | R23 | Symptoms and signs involving the skin and subcutaneous tissue | 11.8 | 9.9 |
| R25 | R29 | Symptoms and signs involving the nervous and musculoskeletal systems | 12.1 | 16.7 |
| R30 | R39 | Symptoms and signs involving the urinary system | 16.2 | 10.4 |
| R40 | R46 | Symptoms and signs involving cognition, perception, emotional state and behaviour | 14.8 | 18.1 |
| R47 | R49 | Symptoms and signs involving speech and voice | 1.1 | 7.4 |
| R50 | R69 | General symptoms and signs | 104.1 | 74.3 |
| R70 | R79 | Abnormal findings on examination of blood, without diagnosis | 3.6 | 3.4 |
| R80 | R82 | Abnormal findings on examination of urine, without diagnosis | 0.5 | 0.9 |
| R83 | R89 | Abnormal findings on examination of other body fluids, substances and tissues, without diagnosis | 0.8 | 0.6 |
| R90 | R94 | Abnormal findings on diagnostic imaging and in function studies, without diagnosis | 3.8 | 9.0 |
| S00 | S09 | Injuries to the head | 14.6 | 14.6 |
| S10 | S19 | Injuries to the neck | 3.0 | 2.5 |
| S20 | S29 | Injuries to the thorax | 5.2 | 1.7 |
| S30 | S39 | Injuries to the abdomen, lower back, lumbar spine and pelvis | 2.5 | 2.4 |
| S40 | S49 | Injuries to the shoulder and upper arm | 4.4 | 3.1 |
| S50 | S59 | Injuries to the elbow and forearm | 10.2 | 4.1 |
| S60 | S69 | Injuries to the wrist and hand | 12.6 | 8.9 |
| S70 | S79 | Injuries to the hip and thigh | 2.7 | 1.2 |
| S80 | S89 | Injuries to the knee and lower leg | 10.2 | 16.9 |
| S90 | S99 | Injuries to the ankle and foot | 14.8 | 17.5 |
| T00 | T07 | Injuries involving multiple body regions | 1.1 | 2.6 |
| T08 | T14 | Injuries to unspecified part of trunk, limb or body region | 24.7 | 28.1 |
| T15 | T19 | Effects of foreign body entering through natural orifice | 2.2 | 5.1 |
| T20 | T25 | Burns and corrosions of external body surface, specified by site | 1.9 | 1.5 |
| T26 | T28 | Burns and corrosions confined to eye and internal organs | 0.3 | 0.0 |
| T29 | T32 | Burns and corrosions of multiple and unspecified body regions | 4.4 | 2.2 |
| T36 | T50 | Poisoning by drugs, medicaments and biological substances | 1.1 | 0.1 |
| T51 | T65 | Toxic effects of substances chiefly nonmedicinal as to source | 1.1 | 5.3 |
| T66 | T78 | Other and unspecified effects of external causes | 9.9 | 26.4 |
| T79 | T79 | Certain early complications of trauma | 1.1 | 4.2 |
| T80 | T88 | Complications of surgical and medical care, not elsewhere classified | 11.3 | 6.4 |
| T89 | T89 | Other specific complications of trauma | 0.3 | 0.1 |
| T90 | T98 | Sequelae of injuries, of poisoning and of other consequences of external causes | 10.7 | 7.8 |
| V01 | X59 | Accident | 0.8 | 2.3 |
| X60 | X84 | Intentional self-harm | 0.0 | 0.2 |
| X85 | Y09 | Assault | 0.5 | 0.0 |
| Y40 | Y84 | Complications of medical and surgical care | 2.7 | 0.2 |
| Z00 | Z13 | Persons encountering health services for examination and investigation | 118.7 | 160.1 |
| Z20 | Z29 | Persons with potential health hazards related to communicable diseases | 237.7 | 133.0 |
| Z30 | Z39 | Persons encountering health services in circumstances related to reproduction | 209.4 | 170.5 |
| Z40 | Z54 | Persons encountering health services for specific procedures and health care | 26.9 | 15.1 |
| Z55 | Z65 | Persons with potential health hazards related to socioeconomic and psychosocial circumstances | 7.4 | 8.2 |
| Z70 | Z76 | Persons encountering health services in other circumstances | 30.0 | 27.2 |
| Z80 | Z99 | Persons with potential health hazards related to family and personal history and certain conditions influencing health status | 0.5 | 10.7 |
