# Supplementary material for: Asylum-seekers in Germany differ from regularly insured in their morbidity, utilizations and costs of care
Source: PLoS One. 2018 May 24;13(5):e0197881. doi: 10.1371/journal.pone.0197881 (PMC5967831; doi:10.1371/journal.pone.0197881)
Supplement: S4 Appendix — (DOCX) [file pone.0197881.s004.docx]

# S4 Appendix: Morbidity, utilization and expenditures by asylum-seekers’ country-group

A ratio above 1.0 indicates that the observed value for a specific country-group is higher than expected based on its age and gender-composition, relative to all asylum-seekers.

- Country group A: Syria, Afghanistan, Iraq, Iran and Eritrea.
- Country group B: Western Balkan states: Albania, Macedonia, Serbia, Kosovo, Montenegro, Bosnia-Herzegovina.
- County group C: All others, predominantly Armenia and the Russian Federation, as well as several African countries, such as Nigeria and Somalia.

**Table D1. Morbidity by country-group (Observed-to-Expected Ratio based on indirect standardization)**

| **#** | **ICD Chapter** | **Group A** | **Group B** | **Group C** |
| --- | --- | --- | --- | --- |
| 1 | Certain infectious and parasitic diseases | 0.89 | 1.16 | 1.24 |
| 2 | Neoplasms | 0.80 | 1.49 | 1.11 |
| 3 | Diseases of the blood and blood-forming organs and certain disorders involving the immune mechanism | 0.92 | 0.88 | 1.36 |
| 4 | Endocrine, nutritional and metabolic diseases | 0.70 | 1.80 | 1.16 |
| 5 | Mental and behavioral disorders | 0.71 | 1.91 | 1.24 |
| 6 | Diseases of the nervous system | 0.65 | 1.78 | 1.45 |
| 7 | Diseases of the eye and adnexa | 1.02 | 0.75 | 1.13 |
| 8 | Diseases of the ear and mastoid process | 0.99 | 1.34 | 0.79 |
| 9 | Diseases of the circulatory system | 0.64 | 2.04 | 1.09 |
| 10 | Diseases of the respiratory system | 0.88 | 1.69 | 0.82 |
| 11 | Diseases of the digestive system | 0.83 | 1.26 | 1.35 |
| 12 | Diseases of the skin and subcutaneous tissue | 0.98 | 1.14 | 0.97 |
| 13 | Diseases of the musculoskeletal system and connective tissue | 0.93 | 1.18 | 1.07 |
| 14 | Diseases of the genitourinary system | 0.95 | 1.11 | 1.07 |
| 15 | Pregnancy, childbirth and the puerperium | 1.19 | 0.48 | 0.86 |
| 16 | Certain conditions originating in the perinatal period | 1.22 | 1.30 | 0.39 |
| 17 | Congenital malformations, deformations and chromosomal abnormalities | 0.96 | 1.55 | 0.66 |
| 18 | Symptoms, signs and abnormal clinical and laboratory findings, not elsewhere classified | 0.92 | 1.20 | 1.10 |
| 19 | Injury, poisoning and certain other consequences of external causes | 1.04 | 0.84 | 0.98 |
| 21 | Factors influencing health status and contact with health services | 1.02 | 1.00 | 0.94 |

**Table D2. Utilization by country-group (Observed-to-Expected Ratio based on indirect standardization)**

| **Indicator** | **Group A** | **Group B** | **Group C** |
| --- | --- | --- | --- |
|  |  |  |  |
| *Inpatient care* |  |  |  |
| All hospitalizations (per 1,000 insured) | 0.91 | 1.18 | 1.14 |
| Emergency department (per 1,000 insured) | 0.93 | 1.17 | 1.10 |
| Individuals with 1 or more hospitalizations (per 1,000 insured) | 0.94 | 1.03 | 1.15 |
| Hospital days (per 1,000 insured) | 0.77 | 1.24 | 1.48 |
| Average length of stay | 0.85 | 1.05 | 1.29 |
| Individuals with 1 or more hospitalizations related to mental health (per 1,000 insured) | 0.60 | 1.94 | 1.77 |
|  |  |  |  |
| *Outpatient care** |  |  |  |
| Outpatient encounters (per 1,000 insured) | 1.00 | 0.95 | 1.05 |
| Individuals with 1 or more outpatient encounters (per 1,000 insured) | 1.01 | 1.01 | 0.97 |
| Average outpatient encounters, for those who had 1 or more encounters | 0.99 | 0.94 | 1.09 |
| Primary care physician encounters (per 1,000 insured) | 0.98 | 1.06 | 1.00 |
| Specialist encounters (per 1,000 insured) | 1.00 | 0.90 | 1.06 |
| Psychotherapy encounters (per 1,000 insured) | 0.93 | 1.54 | 0.80 |
|  |  |  |  |
| *Drugs and medical equipment* |  |  |  |
| Prescriptions (per 1,000 insured) | 0.93 | 1.10 | 1.14 |
| Insured with 1 or more prescriptions (per 1,000 insured) | 0.98 | 1.04 | 1.05 |
| Average prescriptions per insured for those with 1 or more prescriptions | 0.95 | 1.06 | 1.09 |
| Prescribed medical equipment (per 1,000 insured) | 1.02 | 2.52 | - |
|  |  |  |  |
| *Dental care** |  |  |  |
| Dental encounters (per 1,000 insured) | 1.04 | 1.23 | 0.69 |
| Dental encounters for prostheses (per 1,000 insured) | 1.27 | 0.92 | 0.31 |
| Individuals with 1 or more dental encounters (per 1,000 insured) | 1.03 | 1.20 | 0.71 |
| Average dental encounters for those with 1 or more dental encounters | 1.00 | 1.02 | 0.97 |

*The above figures are not annual because the length of the underlying insurance spells may be shorter. However, the columns are comparable because the spells for each match are identical.
* Data for outpatient and dental care are for quarters 1-3 of 2016 only.*

**Table D3. Average expenditures (annualized)**

|  | **Group A** | **Group B** | **Group C** | **Asylum- seekers** | **Matched  comparison** |
| --- | --- | --- | --- | --- | --- |
| Hospital | € 857 | € 1,092 | € 1,413 | € 1,013 | € 435 |
| Outpatient* | € 310 | € 225 | € 324 | € 305 | € 358 |
| Drugs | € 108 | € 374 | € 296 | € 220 | € 402 |
| Dental care* | € 148 | € 223 | € 119 | € 153 | € 304 |
| Other | € 173 | € 250 | € 183 | € 194 | € 221 |
| **Total** | **€ 1,596** | **€ 2,165** | **€ 2,336** | **€ 1,884** | **€ 1,719** |

** Outpatient and dental care expenditures extrapolated from Q1-Q3 to full year*
