# Supplementary material for: Asylum-seekers in Germany differ from regularly insured in their morbidity, utilizations and costs of care
Source: PLoS One. 2018 May 24;13(5):e0197881. doi: 10.1371/journal.pone.0197881 (PMC5967831; doi:10.1371/journal.pone.0197881)
Supplement: S5 Appendix — (DOCX) [file pone.0197881.s005.docx]

# S5 Appendix: Selected case studies

**Table E.1: Prevalence, expenditures and prescriptions for three case studies**

| **Condition and Group** | **Prevalence  (per 1,000 insured)** | **Expenditures (€, per diagnosed)** | | **Prescriptions (per diagnosed)** | |
| --- | --- | --- | --- | --- | --- |
|  |  | **Hospital** | **Pharma-ceuticals** | **Prescriptions** | **Defined daily dose (DDD)** |
| *Acute inflammation of the mucous membranes in ear, nose and throat* | | | | | |
| Asylum-seekers | 110 | 841 | 127 | 4.2 | 87 |
| Matched comparison | 90 | 551 | 629 | 4.7 | 156 |
| *Hypertension* | | | | | |
| Asylum-seekers | 46 | 2,740 | 525 | 6.4 | 434 |
| Matched comparison | 36 | 913 | 665 | 7.3 | 567 |
| *Psychological disorders* | | | | | |
| Asylum-seekers | 146 | 1,870 | 262 | 4.2 | 182 |
| Matched comparison | 122 | 1,452 | 519 | 5.1 | 262 |

ICD codes used for the three conditions

| **Condition** | **ICD odes from outpatient or primary inpatient diagnosis** |
| --- | --- |
| Acute inflammation of the mucous membranes in ear, nose and throat | J00, J01, J02.8, J02.9, J03.8, J03.9, J04, J05, J06, J36 |
| Hypertension | I10-I15 |
| Psychological disorders | F00-F99 |
